# The Potential of Horizontal Wells for Aquifer Storage and Recovery in Saline Aquifers

**DOI:** 10.1111/gwat.70054

**Published:** 2026-02-27

**Authors:** Simon Kreipl, Mark Bakker, Boris M. van Breukelen

**Affiliations:** ^1^ Department of Water Management, Faculty of Civil Engineering and Geosciences Delft University of Technology Delft The Netherlands

## Abstract

Aquifer Storage and Recovery (ASR) is a managed aquifer recharge method where water is injected and later extracted using wells. In saline aquifers, ASR performance is often limited by dispersive mixing, which creates a transition zone at the edge of the injected freshwater and buoyancy‐driven flow, which causes the freshwater to rise and deform during storage—both reducing recovery efficiency. This study investigates whether horizontal wells can improve ASR performance in saline, low‐transmissivity aquifers by achieving acceptable recovery efficiencies and outperforming conventional vertical wells. Three configurations were evaluated numerically with MODFLOW 6: a horizontal well, a fully penetrating vertical well, and a dual well system with a fully penetrating injection well and a partially penetrating extraction well. Models were tested on a large set of parameter combinations from Latin Hypercube Sampling, targeting conditions where vertical wells perform poorly. The horizontal well generally achieved higher recovery efficiencies, with a median of 45% after five ASR cycles, compared to 6% and 16% for the fully and partially penetrating vertical wells. Its advantage was greatest under strong buoyancy conditions, where vertical wells failed to recover any freshwater. While dispersive mixing reduced horizontal well performance by causing earlier saltwater breakthrough, it improved vertical well recovery by stabilizing the injected freshwater. In conclusion, horizontal wells are promising for ASR when hydraulic conditions require multiple vertical wells and when buoyancy‐driven flow significantly limits vertical well performance.

## Introduction

Groundwater resources are facing depletion in many parts of the world. Managed aquifer recharge offers a valuable solution by enhancing groundwater recharge and storage through human intervention (Dillon et al. 2019; Scanlon et al. 2023). Aquifer Storage and Recovery (ASR) is one type of managed aquifer recharge, where wells are used for the injection and extraction of water. In ASR, water is stored in times of surplus, for later recovery during times of scarcity. In temperate climate zones, such as in the Netherlands, ASR can be used to store excess precipitation in winter, for times of increased demand in dryer summers. In the coastal regions of the Netherlands, brackish and saline aquifers are often the only available and technically feasible storage zones for ASR. Although generally less favorable, applications of ASR in brackish and saline aquifers have been reported (van Ginkel et al. 2014; Zuurbier et al. 2014; Maliva et al. 2020).

A typical ASR cycle consists of three phases: injection, storage, and extraction, each characterized by specific flow and transport processes. During injection, the ambient groundwater is displaced by the recharged water, forming a bubble of freshwater around the well. Dispersive mixing creates a transition zone at the edges of the bubble, with a salinity gradient from freshwater to saline groundwater (Esmail and Kimbler 1967; Ward et al. 2007). In the storage phase, the freshwater bubble deforms due to buoyancy‐driven flow, as the lighter freshwater tends to rise and float on top of the denser groundwater (Ward et al. 2007; Bakker 2010). During extraction, flow reverses and the freshwater bubble contracts as water is withdrawn. Extraction is usually terminated when the extracted water concentration exceeds a specific water quality threshold such as a salinity limit. The recovery efficiency—defined as the percentage of injected water that can be recovered to meet this threshold—is an important metric to evaluate the performance of ASR systems.

The recovery efficiency is higher for high injection volumes, and low hydraulic conductivity, aquifer thickness, and groundwater salinity. These are the key variables that govern buoyancy‐driven flow (e.g., Merritt 1986; Lowry and Anderson 2006; Bakker 2010). A high hydrodynamic dispersion, as a proxy for small‐scale aquifer heterogeneity, increases mixing between water sources. Generally, a wider mixing zone leads to an earlier breakthrough of saltwater during extraction, which reduces the recovery efficiency (e.g., Merritt 1986; Lowry and Anderson 2006; Maliva et al. 2020). A wider mixing zone can also attenuate buoyancy‐driven flow and stabilize the freshwater bubble. This can lead to a net increase in recovery efficiency under some conditions (Ward et al. 2007).

One method to mitigate the adverse impacts of buoyancy‐driven flow is the use of multiple partially penetrating wells (Zuurbier et al. 2014, 2016; Witt et al. 2021). This technique involves using multiple wells within a single borehole, each screened at different depths. When the deeper wells begin to salinize as the fresher water floats upwards, they are successively deactivated, while the shallower wells continue extracting the fresher water. This way, Zuurbier et al. (2014) were able to increase the recovery efficiency from between 15% and 30% to 40% for agricultural use.

The hydraulic conductivity and aquifer thickness (i.e., transmissivity) also determine how much pressure is exerted on the aquifer during injection. If the transmissivity is too low, excessive pressure poses the risk of fracturing confining layers. This limits the range of feasible injection rates for the ASR operation. The infiltrated water then needs to be distributed over multiple wells.

Horizontal directionally drilled wells, initially developed in the oil‐and‐gas industry, were introduced to the groundwater sector for contaminated site remediation in the 1990s (Houben et al. 2022). They have since been used for mine dewatering (Struzina et al. 2011), abstraction of potable water (Sass and Treskatis 2000a, 2000b), and managed aquifer recharge (Zuurbier et al. 2015; Perdikaki et al. 2022). A valuable advantage of these wells is that the injection pressure is distributed over a larger area, making it less pronounced (Steward and Jin 2003). This makes horizontal wells particularly beneficial in low‐transmissivity aquifers, where much greater water volumes can be infiltrated and extracted from a single well, avoiding the need for multiple vertical wells (Beljin and Losonsky 1992; Sass and Treskatis 2000a, 2000b). Additionally, a more evenly distributed drawdown during freshwater extraction in saline aquifers reduces upconing of the fresh‐saltwater interface, increasing the volume of extractable water (Stoeckl and Houben 2012; Pauw et al. 2016).

Currently, horizontal directionally drilled wells may be installed with high positional accuracy (Licht et al. 2001). Nevertheless, horizontal wells remain technically more complex to construct than vertical wells, particularly in unconsolidated sediments. The installation of an effective gravel pack along the horizontal section is challenging. In addition, drilling fluids are typically required to stabilize the borehole during drilling, which necessitates extensive well development and increases the likelihood of skin formation (Houben et al. 2022). Horizontal wells also generally require higher initial capital investment due to the increased complexity of drilling operations and the limited availability of specialized drilling contractors. However, these higher installation costs may be offset by higher well yields and the potential to replace multiple vertical wells with a single horizontal well. In cases where one horizontal well substitutes several vertical wells, system‐level operational costs may be reduced due to the use of a single pump and simplified associated conveyance infrastructure, resulting in lower power supply requirements.

Despite their potential advantages, the application of horizontal wells in ASR is sparsely researched. A first application for agricultural water supply involved installing two superimposed wells, each 70 m in length (Zuurbier et al. 2015). A shallow well, positioned within a freshwater lens in a brackish coastal aquifer, injected and extracted water. A deeper well, positioned below the freshwater lens, continuously extracted brackish water to counteract upconing during freshwater extraction from the shallow well, and maintain the position of the fresh–saltwater interface during the ASR operation. Under these specific conditions, horizontal wells showed promising results (Zuurbier et al. 2015).

These findings suggest that horizontal wells could facilitate ASR installations in conditions previously considered infeasible, particularly low‐transmissivity aquifers. Furthermore, horizontal wells may achieve higher recovery efficiencies in saline aquifers because they may reduce the effects of buoyancy‐driven flow and reduce upconing. However, the flow and transport processes that govern the performance of horizontal well ASR systems have not yet been investigated. Additionally, no studies have compared the recovery efficiency of horizontal well ASR systems with conventional vertical wells.

This study investigates the potential of horizontal wells for ASR in saline aquifers, particularly under conditions where vertical wells do not perform well. The objective is to assess whether horizontal wells can achieve operationally acceptable recovery efficiencies in such settings and to identify if and when they may offer advantages over vertical wells. The study aims to develop a generalized understanding of how buoyancy‐driven flow and dispersive mixing influence ASR performance through numerical modeling of density‐dependent groundwater flow and solute transport.

## Methods

This study employs numerical modeling of density‐dependent groundwater flow and solute transport to evaluate the potential advantages and limitations of horizontal wells for ASR in saline aquifers. Simulations are performed on a conceptual groundwater system comprising of a homogeneous storage aquifer, confined above and below by horizontal, impermeable layers. The aquifer is characterized by its thickness b, horizontal and vertical hydraulic conductivity $k_{h}$ and $k_{v}$, and a background groundwater salinity $c_{\mathrm{GW}}$, expressed as total dissolved solids (TDS). Changes in aquifer storage due to compressibility (specific storage) are neglected. Transport processes are governed by the aquifer's effective porosity $n_{\mathrm{eff}}$, longitudinal and transversal dispersivity $\alpha_{L}$ and $\alpha_{T}$, and molecular diffusion $D_{m}$.

**Figure 1:**
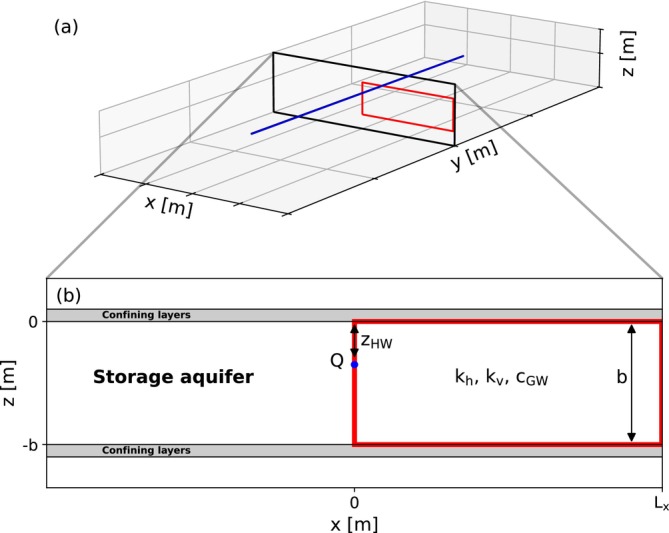
(a) Three‐dimensional conceptual model of an infinitely long horizontal well; (b) Two‐dimensional cross‐section along *x*‐direction of a slice of the horizontal well. The blue pipe represents the horizontal well. The red box represents the domain of the numerical model.

**Figure 2:**
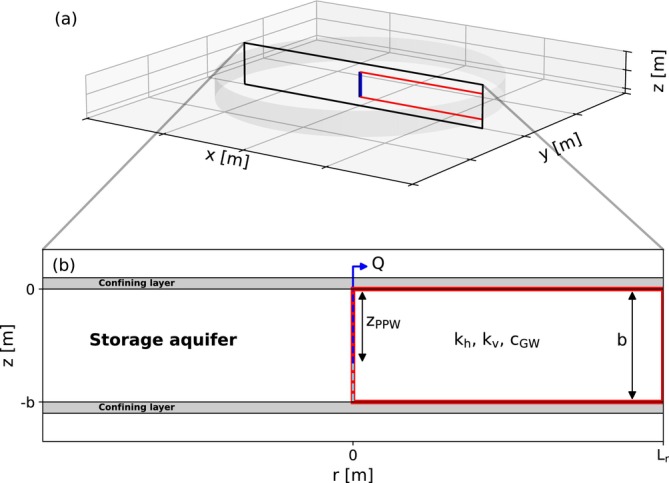
(a) Three‐dimensional conceptual model of a vertical well; (b) Radial cross‐section through the center of the vertical well. The blue pipe represents the vertical well. The red box represents the domain of the numerical model.

Two separate models are developed: one representing a horizontal well and the other one a vertical well. The horizontal well is $L_{\mathrm{HW}}$ long, as presented in Figure 1a. For this study, a two‐dimensional vertical cross‐section is simulated, as shown in Figure 1b; tip effects are not considered here. The well has a radius $r_{\mathrm{HW}}$ and is positioned near the top of the storage aquifer at a depth $z_{\mathrm{HW}}$. The conceptual model of the vertical well system is depicted in Figure 2a. A radial cross‐section is simulated, as shown in Figure 2b. The well has a radius $r_{\mathrm{VW}}$. Two configurations of the vertical well system are analyzed: (i) A fully penetrating vertical well (Vertical‐FP) which fully penetrates the aquifer thickness, facilitating both injection and extraction through the same well; (ii) a partially penetrating vertical well (Vertical‐PP) which is a dual‐well configuration comprising of a fully penetrating injection well and a partially penetrating extraction well. The latter extends from the top of the aquifer to a depth $z_{\mathrm{PPW}}$. Both wells are in the same location and are screened in the same borehole.

Multiple ASR cycles of 360 days ($t_{\text{CYCLE}}$) are simulated, each comprising of three main phases: a 90 day injection phase ($t_{\mathrm{INJ}}$) during which freshwater with a TDS concentration $c_{\mathrm{FW}}$ is injected; a 180 day storage phase ($t_{\mathrm{STO}}$); and an extraction phase that continues as long as the TDS concentration of the extracted water $c_{\mathrm{EXT}}$ remains below a predefined maximum threshold $c_{\mathrm{MAX}}=650\;\mathrm{mg}/L$—a value commonly used for potato irrigation. Once this limit is reached, the system enters an idle phase until the start of the next cycle. If the limit is not reached, extraction stops after 90 days. The discharge from the well Q is identical during the injection and extraction phases. For the horizontal well model, the well discharge is divided over the length of the horizontal well as $Q_{\mathrm{HW}}=Q/L_{\mathrm{HW}}$. The performance of the respective systems is evaluated based on the recovery efficiency, defined as the percentage of injected water with a concentration less than $c_{\mathrm{MAX}}$ that can be recovered. Numerical values for the hydrogeological conditions and design and operation choices are summarized in Table 1.

**Table 1:** Hydrogeological Conditions and Design and Operation Choices.

Hydrogeological Conditions	Symbol	Unit	Value
Aquifer thickness	b	m	5–25
Horizontal hydraulic conductivity	$k_{h}$	m/d	10–50
Anisotropy ratio for conductivity	$k_{h}/k_{v}$	‐	5 and 10
Background groundwater salinity	$c_{\mathrm{GW}}$	mg/L	1000–35,000
Effective porosity	$n_{\mathrm{eff}}$	‐	0.3
Longitudinal dispersivity	$\alpha_{L}$	m	0.1 and 0.5
Anisotropy ratio for dispersivity	$\alpha_{T}/\alpha_{L}$	‐	0.1
Molecular diffusion	$D_{m}$	m^2^/d	$8.64\times 10^{-5}$
**Design and Operation Choices**			
Well radius	$r_{\mathrm{HW}}$, $r_{\mathrm{VW}}$	m	0.25, 0.15
Depth of horizontal well	$z_{\mathrm{HW}}$	m	2
Length of horizontal well	$L_{\mathrm{HW}}$	m	200
Depth of PPW	$z_{\mathrm{PPW}}$	m	2/3⋅b
Well discharge	Q	m^3^/d	100–1000
Cycle length	$t_{\text{CYCLE}}$	d	360
Injection period length	$t_{\mathrm{INJ}}$	d	90
Storage period length	$t_{\mathrm{STO}}$	d	180
Injected freshwater concentration	$c_{\mathrm{FW}}$	mg/L	0
Maximum extraction concentration	$c_{\mathrm{MAX}}$	mg/L	650

### Numerical Model

Numerical modeling of groundwater flow and solute transport was conducted using MODFLOW 6, Version 6.6.1. (Hughes et al. 2017). MODFLOW 6 enables coupling of a groundwater flow model (Langevin et al. 2017) with a solute transport model (Langevin et al. 2022). Density‐dependent flow is incorporated using the BUY‐package (Langevin et al. 2020), which accounts for buoyancy effects. Advection is simulated using a second‐order Total Variation Diminishing (TVD) scheme. Model development, running, and post‐processing were carried out using the FloPy Python package (Bakker et al. 2016). Computationally intensive simulations were performed on the DelftBlue Supercomputer (DHPC 2022).

**Figure A1:**
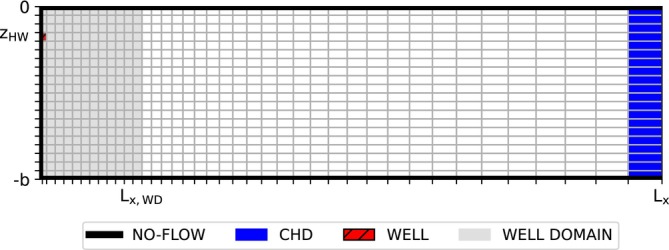
Two‐dimensional cross‐section of the numerical model for the horizontal well.

**Figure A2:**
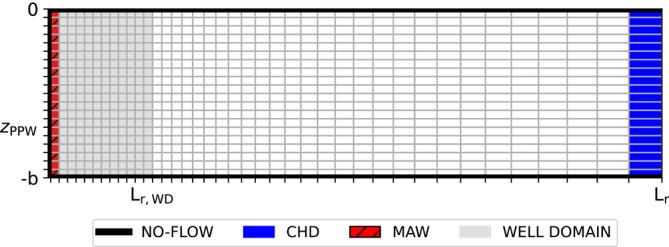
Radial cross‐section of the numerical model for the vertical well.

The model for the horizontal well system is shown in Figure A1. One half of the flow domain is simulated to reduce computational effort. The well is positioned at x=0m, where a no‐flow boundary is applied. The horizontal well is simulated with the MODFLOW WEL‐package and is represented using a single model cell. The model for the vertical well system is shown in Figure A2. An axisymmetric model is used for the vertical well as described by Langevin (2008). The well is positioned at r=0m. The vertical well is simulated using the MODFLOW Multi‐Aquifer‐Well‐package (MAW), where the specified well discharge is distributed by maintaining a hydrostatic hydraulic head over the connected MAW‐cells. For both system types, the upper and lower boundaries of the model are defined as no‐flow boundaries and a constant‐head (CHD) boundary with h=0m is applied at the outer model edges $L_{x}$ and $L_{r}$, which are sufficiently distant to avoid influencing the simulation results.

Rectangular grid cells are used for both models. A refined grid is used in the vicinity of the well—referred to here as the well domain—extending up to a distance of $L_{x,\mathrm{WD}}$ and $L_{r,\mathrm{WD}}$, respectively. This region is discretized using uniform cells with a width of Δx and Δr. For the horizontal well model, the first column of cells in the x‐direction has a width of Δx/2 because only half of the domain is modeled. Beyond the well domain, horizontal cell sizes increase gradually by a factor of 1.05 to reduce computational demand while maintaining accuracy. Vertically, the aquifer is discretized into layers with a uniform thickness of Δz. The time step size Δt is dynamically adjusted during the simulation to satisfy a Courant number of 1.4, with a maximum allowable step size of 1 day. The parameters used in the numerical model are summarized in Table 2.

**Table 2:** Model Parameters Used in MODFLOW 6.

Flow and Transport Solver Settings	Symbol	Value
Maximum outer iterations	outer_maximum	30
Maximum inner iterations	inner_maximum	100
Head change convergence criterion (outer)	outer_dvclose	$1\times 10^{-4}$ m
Head change convergence criterion (inner)	inner_dvclose	$1\times 10^{-4}$ m
Linear acceleration method	linear_acceleration	“BICGSTAB”
Relaxation factor	relaxation_factor	0.97
**Advection Solver Settings**		
Advection solver	scheme	TVD
Courant number	ats_percel	1.4
**Spatial Discretization**		
Model domain length in *x*‐/*r*‐direction	$L_{x}$, $L_{r}$	1024.80, 1025.05
Well domain length in *x*‐/*r*‐direction	$L_{x,\mathrm{WD}}$, $L_{r,\mathrm{WD}}$	100
Vertical cell size	Δz	0.5 m
Number of layers	$n_{\mathrm{LAY}}$	b/Δz
Horizontal cell size	Δx, Δr	0.5 m
Number of horizontal cells	$n_{\mathrm{HC}}$	294
Number of well cells	$n_{\mathrm{WC}}$	1 (Horizontal)
		b/Δz (FP)
		$z_{\mathrm{PPW}}/\Delta z$ (PP)

### Selection of Parameter Sets

The effect of six parameters on ASR performance is investigated: aquifer thickness b, horizontal hydraulic conductivity $k_{h}$, background groundwater salinity $c_{\mathrm{GW}}$, well discharge Q, longitudinal dispersivity $\alpha_{L}$ (while $\alpha_{T}=0.1\alpha_{L}$), and vertical anisotropy of the hydraulic conductivity $k_{h}/k_{v}$. The first four parameters are varied over a range for one value of $\alpha_{L}$ and $k_{h}/k_{v}$, after which simulations are repeated with the same set of parameters but a different value of $\alpha_{L}$ and a different value of $k_{h}/k_{v}$. Parameter sets of the four varied parameters are based on the parameter D introduced by Bakker (2010) as

(1) $$D=\frac{Q}{k_{h}\nu b^{2}}$$

where ν is the density difference ratio [−] as

(2) $$\nu =\frac{\rho_{\mathrm{GW}}-\rho_{\mathrm{FW}}}{\rho_{\mathrm{GW}}}$$

where $\rho_{\mathrm{GW}}$ and $\rho_{\mathrm{FW}}$ are the density of the background groundwater and injected freshwater, respectively [$\mathrm{kg}/m^{3}$]. The density of groundwater is obtained from the salinity using the approximate formula

(3) $$\rho_{\mathrm{GW}}=\rho_{\mathrm{FW}}+\frac{\partial \rho }{\partial C}\cdot \left(c_{\mathrm{GW}}-c_{\mathrm{FW}}\right)$$

where $\rho_{\mathrm{FW}}=1000\;\mathrm{kg}/m^{3}$ and ∂ρ/∂C is the density slope which for the range from freshwater to seawater is approximated as 0.734 for concentrations in TDS. Note that all computations are done in $\mathrm{kg}/m^{3}$, but the concentrations are reported as mg/L in this paper.

The parameter D incorporates the key variables that influence buoyancy‐driven flow. Low values of D are associated with lower recovery efficiencies, while higher values—resulting, for example, from higher discharge rates or lower hydraulic conductivity, background salinity, or aquifer thickness—tend to yield higher recovery efficiencies. In this study, D is used as a proxy for buoyancy‐driven flow. By varying the parameters that comprise D, the influence of buoyancy on the recovery efficiency of the horizontal and vertical well models is investigated. The recovery efficiencies reported by Bakker (2010) are based on a Dupuit interface model for a vertical well which did not take into account dispersive mixing or vertical anisotropy of the hydraulic conductivity. For Dupuit interface flow, the recovery efficiency is a function only of the parameter D and of the relative lengths of the injection, storage, and recovery periods. In this study, the effect of mixing and vertical anisotropy of the hydraulic conductivity on the recovery efficiency is investigated.

The analysis is focused on low‐transmissivity aquifers where vertical wells do not perform well. The ranges of the varied parameters are selected to reflect these conditions. The horizontal hydraulic conductivity values range from 10 to 50 m/d for fine‐ to medium‐grained sands (Domenico and Schwartz 1990). Aquifer thickness values range from 5 to 25 m to represent relatively thin aquifers. The background groundwater salinity range is from 1000 to 35,000 mg/L TDS, covering the spectrum from brackish to seawater. The total injection and extraction rates range from 100 to 1000 $m^{3}/d$. For the horizontal well model, the discharge is distributed uniformly along the well. For the vertical well model, the discharge is distributed uniformly along the well screen so that the pressure is hydrostatic in the well.

A total of 250 parameter sets are generated using Latin Hypercube Sampling (McKay et al. 1979) based on the ranges specified in Table 1 (with $\alpha_{L}=0.1\;m$ and $k_{v}=k_{h}/5$). The parameter sets are filtered using the following three constraints to avoid parameter combinations that are not physically plausible or do not align with the scope of this investigation:

1. D‐value: D>1
2. Transmissivity: $T=k_{h}b<1000\;m^{2}/d$
3. Injection pressure: s<5m

Parameter sets with D<1 are excluded because buoyancy effects are considered too large. Sets with $T>1000\;m^{2}/d$ are excluded to focus the study on hydraulically restricted aquifers. Finally, sets with s>5m are removed due to the risk of fracturing confining layers. The injection pressure is calculated for a single, fully penetrating vertical well following Theis (1935) (e.g., as presented in Bakker and Post 2022), with a specific storage coefficient ($S_{s}$) of $10^{-4}\;m^{-1}$.

**Figure 3:**
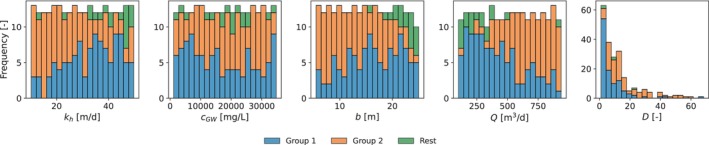
Frequency distribution of parameters. Group 1: Parameter sets that meet all three constraints ($n_{1}=109$). Group 2: Parameter sets that meet D‐value and transmissivity constraints but not the injection pressure constraint ($n_{2}=122$). Rest: Parameter sets that do not meet any constraints.

The frequency distributions of the four varied input variables as well as parameter D are shown in Figure 3. The frequency of each parameter is roughly evenly distributed across the specified range. The D‐values resulting from the generated parameter sets are predominantly low: 136 out of 250 samples result in D<10. This indicates that, given the input ranges, most combinations correspond to scenarios with relatively strong buoyancy‐driven flow and potentially lower recovery efficiencies. Of 250 sets, 236 meet the D‐value constraint. As has been discussed, this constraint excludes combinations of low well discharge and high hydraulic conductivity, background salinity, or aquifer thickness. Of 250 sets, 242 meet the transmissivity constraint. This constraint excludes combinations of high aquifer thickness and hydraulic conductivity. Of 250 sets, 128 meet the injection pressure constraint. This constraint excludes parameter sets with high well discharge combined with low aquifer thickness and hydraulic conductivity. Two groups of parameter sets are created. The blue bars show the frequency distributions of the first group of parameter sets that meet all three constraints. This group contains 109 parameter sets. The orange bars show the frequency distribution of the second group of parameter sets that meet only the D‐value and transmissivity constraint but not the injection pressure constraint. This group contains 122 sets consisting mainly of combinations of high well discharge and low aquifer thickness and hydraulic conductivity. The green bars show the frequency distribution of the remainder of the original 250 parameter sets that do not meet any of the constraints.

The first group of parameter sets represents the subset of physically plausible scenarios that align with the scope of this investigation. These parameter sets are used as input variables for the horizontal and vertical well models to assess whether horizontal wells can achieve operationally feasible recovery efficiencies and to identify if and when they offer advantages over vertical wells. These simulations are repeated once with $\alpha_{L}$ increased from 0.1 to 0.5 m, and once for $k_{v}$ reduced from $k_{h}/5$ to $k_{h}/10$. The second group of parameter sets is used as input variables for only the horizontal well model because the injection pressure is too high for a vertical well. Finally, two additional investigations are performed on the first group of parameter sets to evaluate how the simulation results are effected by more accurate solutions. This includes (i) a comparison with a model that uses a higher order TVD‐scheme for the simulation of advective transport, and (ii) a comparison with a finer grid resolution to reduce numerical dispersion. In some of the investigations, one or two simulations failed to converge for the vertical well configuration. These cases were excluded across all well configurations to ensure consistency, and the analysis was performed on the remaining successful simulations.

## Results and Discussion

### Flow and Transport Dynamics

The flow and transport dynamics of an ASR system are illustrated using a representative example simulation for the first ASR cycle in a brackish aquifer. The parameters of the selected example simulation are listed in Table 3.

**Table 3:** Parameters of Example Simulation (Simulation Number = 32).

Parameter	Symbol	Value
Aquifer thickness	b	21.5 m
Horizontal hydraulic conductivity	$k_{h}$	41.7 m/d
Background groundwater salinity	$c_{\mathrm{GW}}$	6,100 mg/L
Well discharge	Q	522.9 m^3^/d
D‐value	D	6.06

**Figure 4:**
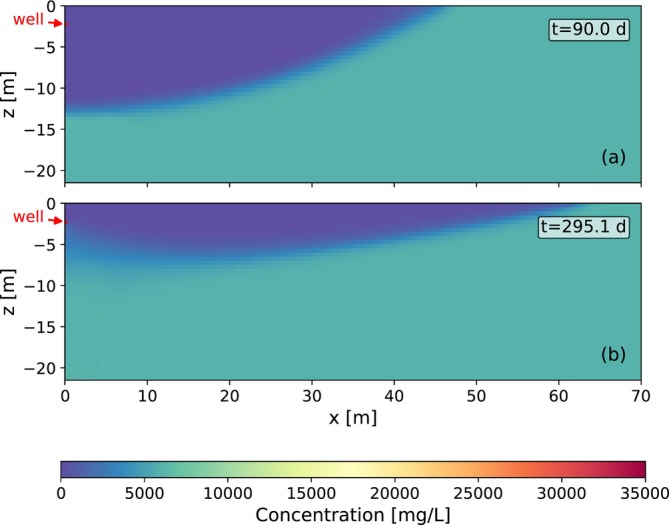
Concentration distribution for a horizontal well (a) at the end of injection (after 90 days) and (b) at the end of extraction (when $c_{\mathrm{EXT}}=c_{\mathrm{MAX}}$) for the example simulation. Due to symmetry, only one half of the flow domain is shown. The red area with the black lines on the left of the figure is the well cell (an annotation arrow labeled “well” indicates its position).

**Figure 5:**
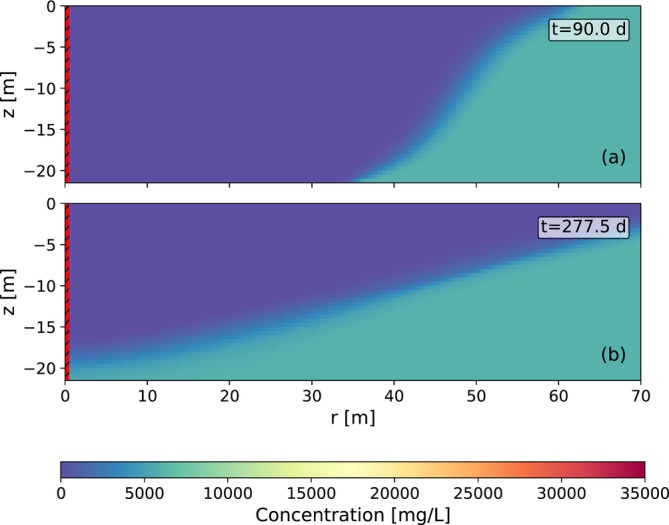
Concentration distribution for a fully penetrating vertical well (a) at the end of injection (after 90 days) and (b) at the end of extraction (when $c_{\mathrm{EXT}}=c_{\mathrm{MAX}}$) for the example simulation. A radial cross‐section is shown. The red area with black lines on the left of the figure is the well.

During ASR, the injection of freshwater displaces ambient groundwater, forming a freshwater bubble around the well. A mixing zone develops between the injected freshwater and the background groundwater. A cross‐section of the concentration distribution of the example simulation is shown in Figure 4 for the horizontal well and in Figure 5 for the vertical well. Concentrations are presented at (a) the end of injection and (b) the end of extraction (when $c_{\mathrm{EXT}}=c_{\mathrm{MAX}}$), respectively. With the horizontal well, the injected freshwater moves downward and outward, forming a horizontally stretched semi‐cylinder. Over time, buoyancy effects push the freshwater further outward at the aquifer top. Upward migration is limited by the presence of an impermeable cover layer. With the vertical well, the freshwater spreads radially, forming a cylindrical bubble. Over time, buoyancy effects cause upward migration of the freshwater, tilting the vertical sides of the cylinder. This leads to an extended bubble radius at the aquifer top and a reduced radius at the bottom.

**Figure 6:**
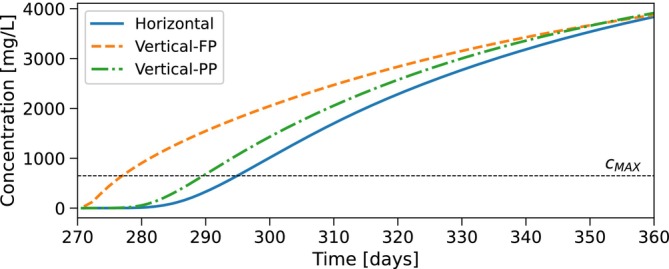
Extracted water concentration during the first ASR cycle for the example simulation. Extraction starts at 270 days. Vertical wells show the flux weighted average concentration across the respective well cells; horizontal wells show concentration from the single well cell.

During extraction, flow reverses and the freshwater bubble contracts as water is withdrawn. The extracted concentrations during the first ASR cycle of the example simulation with the horizontal well and the fully and partially penetrating vertical wells are shown in Figure 6. The concentration begins to rise as the mixing zone reaches the well. For the horizontal well, saltwater is drawn toward the well through upconing, which leads to a continued increase in concentration. Extraction is terminated when the average concentration of extracted water exceeds $c_{\mathrm{MAX}}$. Residual freshwater remains in the upper part of the aquifer. For the example in Figure 6, this occurs after 295.1 days (25.1 days of extraction), yielding a recovery efficiency of 27%. For the fully penetrating vertical well, saltwater first reaches the well at the bottom due to the reduced bubble radius. For the example in Figure 6, extraction is stopped after 277.5 days, resulting in a recovery efficiency of 7%. For the partially penetrating well, the end of extraction is delayed from 277.5 to 290 days as compared to the fully penetrating well, improving recovery efficiency from 7% to 22%.

### Recovery Efficiencies

**Figure 7:**
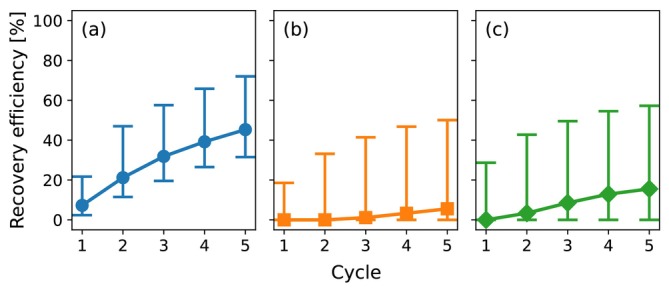
Progression of the recovery efficiency over five ASR cycles. (a) is the horizontal well, (b) is the fully penetrating vertical well, and (c) is the partially penetrating vertical well. Markers indicate the median per cycle; whiskers show the interquartile range.

The simulations for the horizontal well and the fully and partially penetrating vertical wells were repeated for the first group of parameter sets over five ASR cycles. The progression of recovery efficiency across these cycles is presented in Figure 7 for (a) the horizontal well, (b) the fully penetrating vertical well, and (c) the partially penetrating vertical well. The markers represent the median recovery efficiency, while the whiskers represent the interquartile range. Initial recovery efficiencies are low for all systems: the horizontal well starts at a median recovery efficiency of 7% compared to 0% for both vertical well configurations. Recovery efficiency increases with each cycle. This trend results from unrecovered water that remains in the aquifer and becomes available for extraction in subsequent cycles. Recovery efficiency increases until a balance is reached between extractable water and residual storage, at which point a system specific maximum is reached. After five cycles, the horizontal well achieves a median recovery efficiency of 45%, while the fully and partially penetrating vertical wells reach 6% and 16%, respectively. These values are conservative due to the selection of a long storage period (180 days) and a low $C_{\mathrm{MAX}}$ of 650 mg/L. Shorter storage durations or a higher $C_{\mathrm{MAX}}$ will improve recovery efficiency across all systems, while the differences between systems are unlikely to change significantly.

**Figure 8:**
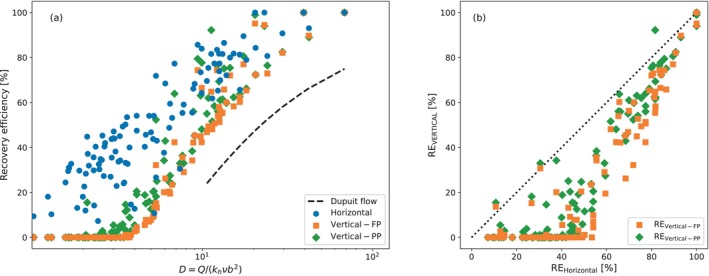
Recovery efficiency after five ASR cycles. (a) is the recovery efficiency as a function of parameter D. “Dupuit flow” refers to recovery efficiency from the Dupuit interface flow model (Bakker 2010). (b) is a comparison of recovery efficiencies of the horizontal well with the fully and partially penetrating vertical wells.

The recovery efficiency after five cycles is shown in Figure 8 for all of the parameter sets of the first group. The recovery efficiency is plotted as a function of the parameter D in Figure 8a. The line marked as “Dupuit flow” refers to the recovery efficiency from the Dupuit interface flow model (Bakker 2010). The recovery efficiency of the horizontal well is compared to the fully and partially penetrating vertical well in Figure 8b. The recovery efficiency is lowest for small D‐values. Among the 50 scenarios with D < 4, the horizontal well achieves a median recovery efficiency of 33%, while the median of the fully and partially penetrating vertical wells is 0%. This highlights the potential of horizontal wells to provide viable recovery under conditions where vertical wells are ineffective. The recovery efficiency increases with D across all configurations until a maximum of 100% is reached. The difference between the horizontal well and the fully and partially penetrating vertical wells decreases with a higher general recovery efficiency.

Significant variability in recovery efficiency is observed for similar D‐values, especially with horizontal wells. This variability arises from the dual role of groundwater salinity: it affects both the density difference (captured in D with the density difference ratio, ν) as well as the degree of mixing at the fresh–saltwater interface (not captured in D). Mixing with a higher salinity groundwater increases the concentration of extracted water more rapidly and yields a lower recovery efficiency compared to another parameter set with a lower groundwater salinity but a similar D‐value because of a higher aquifer thickness or hydraulic conductivity or a lower total discharge.

### Effect of Dispersivity

**Figure 9:**
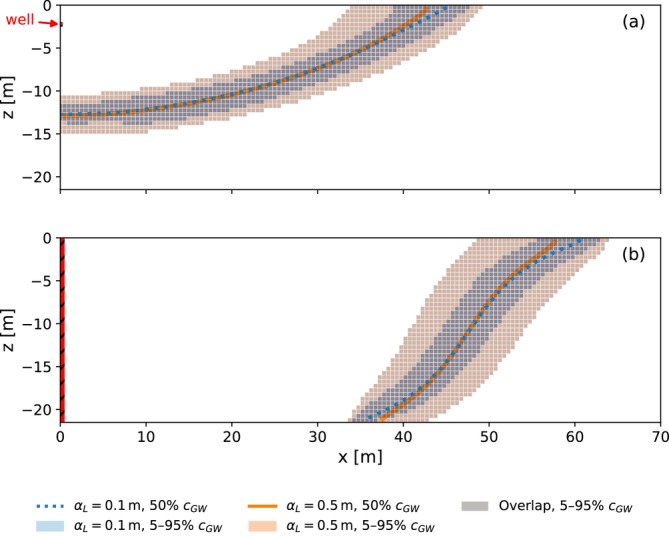
Comparison of mixing zones at the end of injection (*t* = 90 days) for $\alpha_{L}=0.1\;m$ and 0.5m for the example simulation. (a) is the horizontal well and (b) is the fully penetrating vertical well. Shaded area shows 5%–95% concentration of background groundwater, while colored curves represent the 50% contour. The shaded area for $\alpha_{L}=0.5\;m$ completely overlaps the shaded area for $\alpha_{L}=0.1\;m$ in this case.

The simulations of the first group of parameter sets are repeated to investigate the effect of the dispersivity on the ASR simulations. The dispersivity is a model parameter that captures the influence of heterogeneity on solute transport and governs mixing between injected freshwater and ambient groundwater. The longitudinal dispersivity is increased from $\alpha_{L}=0.1\;m$ to 0.5m. A comparison of the mixing zones at the end of injection for the example simulation of Table 3 is given in Figure 9. Both the result for (a) the horizontal well and (b) the fully penetrating vertical well are shown. In these figures, the shaded areas represent 5%–95% concentration of background groundwater, which serve as indicators of the mixing zone width. As expected, the mixing zone becomes wider with increased dispersivity for both systems. The blue and orange contours represent the 50% concentration. A comparison of the contours of the vertical well shows that the interface is slightly more tilted at the aquifer top and bottom when the dispersivity is smaller. This suggests that a wider mixing zone (i.e., larger dispersivity) attenuates the interface tilting process, consistent with findings reported by Ward et al. (2007).

**Figure 10:**
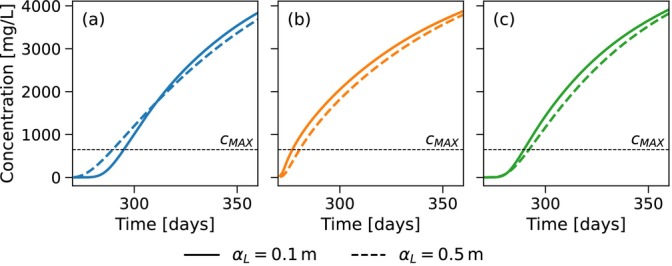
Comparison of the extracted water concentration during the first ASR cycle for $\alpha_{L}=0.1\;m$ and $\alpha_{L}=0.5\;m$ for the example simulation. (a) is the horizontal well, (b) is the fully penetrating vertical well, and (c) is the partially penetrating vertical well. Extraction starts at 270 days. Vertical wells show flux weighted average concentration across the respective well cells; horizontal well shows concentration from the single well cell.

A comparison of the extracted water concentration during the first ASR cycle for the example simulation of Table 3 with the two dispersivities is shown in Figure 10 for (a) the horizontal well, (b) the fully penetrating vertical well, and (c) the partially penetrating vertical well. For the horizontal well, extraction is terminated earlier at 289.8 days for $\alpha_{L}=0.5\;m$ compared to 295.1 days for $\alpha_{L}=0.1\;m$, resulting in a decrease in recovery efficiency from 27% to 22%. This reduction is primarily attributed to the earlier arrival of the saltwater front, driven by the wider mixing zone. In the case of the fully and partially penetrating vertical well, extraction ends slightly later at 281.5 days instead of 277.5 and 292.2 days instead of 290 days, increasing recovery efficiency from 7% to 11% and from 22% to 24%. Here, the earlier saltwater arrival is counter‐balanced by the attenuated interface tilting, resulting in a net increase in recovery efficiency.

**Figure 11:**
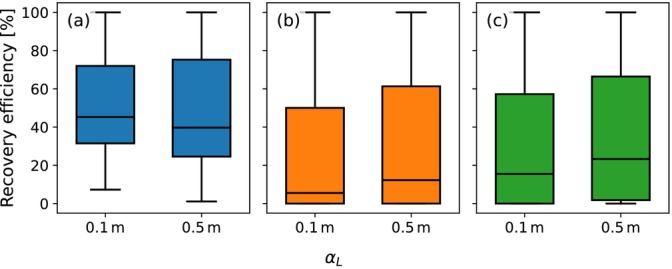
Boxplots of the recovery efficiencies after five ASR cycles for $\alpha_{L}=0.1\;m$ and $\alpha_{L}=0.5\;m$. (a) is the horizontal well, (b) is the fully penetrating vertical well, and (c) is the partially penetrating vertical well.

Boxplots comparing the recovery efficiencies of the two dispersivity values after five ASR cycles for the simulated parameter sets of the first group are shown in Figure 11 for (a) the horizontal well, (b) the fully penetrating vertical well, and (c) the partially penetrating vertical well. For the horizontal well, the 75th percentile recovery efficiency increases slightly from 72% to 75%. In contrast, the median and 25th percentile decrease from 45% to 40% and from 32% to 25%, respectively. These results suggest that while high‐efficiency scenarios remain relatively unaffected by increased mixing, the majority experience a decline in recovery efficiency due to the earlier arrival of the concentration front. For the fully penetrating vertical well, the 25th percentile remains constant at 0%. The median and 75th percentile show increases from 6% to 12% and from 50% to 61%. For the partially penetrating vertical well, the 25th percentile, median, and 75th percentile increase from 0% to 2%, 16% to 23%, and from 57% to 66%, respectively. These trends indicate that vertical wells generally benefit from increased mixing, due to the attenuating effect of a broader mixing zone on interface tilting. In conclusion, an increased degree of dispersive mixing reduces the difference in recovery efficiency between horizontal and vertical well systems.

### Effect of Anisotropy

**Figure 12:**
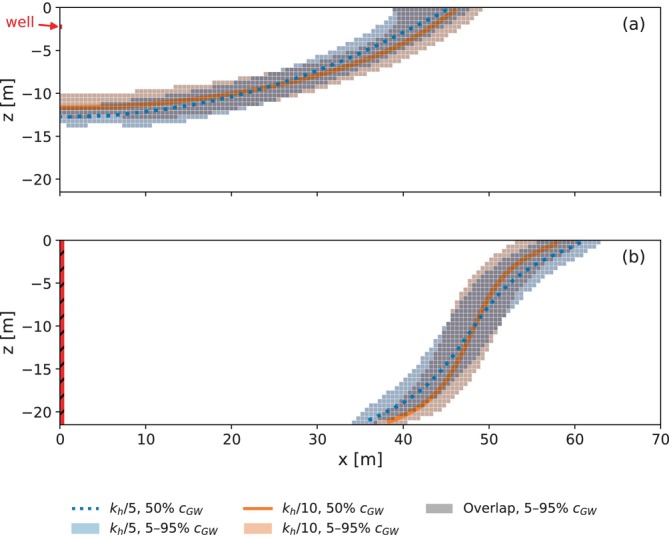
Comparison of mixing zones at the end of injection (*t* = 90 days) for $k_{v}=k_{h}/5$ and $k_{v}=k_{h}/10$ for the example simulation. (a) is the horizontal well and (b) is the fully penetrating vertical well. Shaded area shows 5%–95% concentration of background groundwater, while colored curves represent the 50% contour.

The simulations of the first group of parameter sets are repeated to investigate the effect of the vertical anisotropy of the hydraulic conductivity on the ASR simulations. The vertical hydraulic conductivity is reduced from $k_{v}=k_{h}/5$ to $k_{h}/10$. A comparison of the mixing zones at the end of injection for the example simulation of Table 3 is given in Figure 12. Both the results for (a) the horizontal well and (b) the fully penetrating vertical well are shown. For the horizontal well, it is evident that the freshwater bubble under $k_{v}=k_{h}/10$ extends further laterally but penetrates less deep than in the $k_{v}=k_{h}/5$ case. This behavior results from the reduced vertical hydraulic conductivity, which constrains downward movement and promotes horizontal spreading of the freshwater bubble. For the vertical well, the interface tilting under $k_{v}=k_{h}/10$ is less pronounced as compared to $k_{v}=k_{h}/5$. This is also attributed to the lower vertical hydraulic conductivity which attenuates interface tilting.

**Figure 13:**
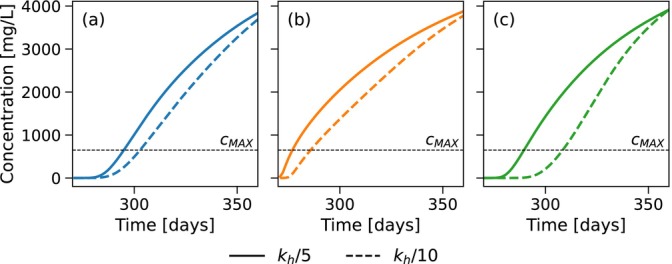
Comparison of the extracted water concentration during the first ASR cycle for $k_{v}=k_{h}/5$ and $k_{v}=k_{h}/10$ for the example simulation. (a) is the horizontal well, (b) is the fully penetrating vertical well, and (c) is the partially penetrating vertical well. Extraction starts at 270 days. Vertical wells show flux weighted average concentration across the respective well cells; horizontal well shows concentration from the single well cell.

A comparison of the extracted water concentration during the first ASR cycle for the example simulation of Table 3 with the two anisotropy values is shown in Figure 13 for (a) the horizontal well, (b) the fully penetrating vertical well, and (c) the partially penetrating vertical well. For the horizontal well, the extraction period is extended from 295.1 days for $k_{v}=k_{h}/5$ to 303.1 days for $k_{v}=k_{h}/10$, resulting in an increase in recovery efficiency from 27% to 36%. For the fully and partially penetrating vertical well, the extraction period is extended from 277.5 to 286.6 days and from 290 to 308.5 days, with recovery efficiency rising from 7% to 17% and from 22% to 42%, respectively. Across all well configurations, the higher anisotropy ($k_{v}=k_{h}/10$) limits vertical flow, thereby reducing the influence of buoyancy. This improves the recovery efficiency by maintaining a more stable freshwater bubble.

**Figure 14:**
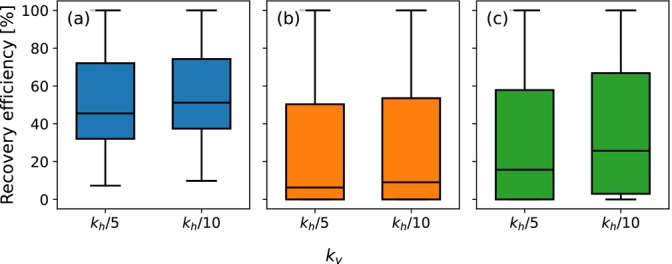
Boxplots of the recovery efficiencies after five ASR cycles for $k_{v}=k_{h}/5$ and $k_{v}=k_{h}/10$. (a) is the horizontal well, (b) is the fully penetrating vertical well, and (c) is the partially penetrating vertical well.

Boxplots comparing the recovery efficiencies of the two anisotropy values after five ASR cycles for the simulated parameter sets of the first group are shown in Figure 14 for (a) the horizontal well, (b) the fully penetrating vertical well, and (c) the partially penetrating vertical well. For the horizontal well, the 25th percentile and median recovery efficiencies increase from 32% to 37% and from 46% to 51%, respectively. The 75th percentile shows a marginal increase from 72% to 74%. These results suggest that the improvements at higher anisotropies are more pronounced in low‐efficiency scenarios, where buoyancy effects are more significant. For the fully penetrating vertical well, the 25th percentile remains unchanged at 0%, while the median and 75th percentile increase from 6% to 9% and from 50% to 54%, respectively. For the partially penetrating vertical well, the 25th, median, and 75th percentiles increase from 0% to 3%, 16% to 26%, and 58% to 67%, respectively. These trends indicate consistent improvements for all of the ASR systems. This improvement is primarily due to the restriction of vertical flow, which mitigates buoyancy‐driven flow.

### High Pressure Conditions

The parameter sets in the second group meet the D‐value and transmissivity constraints, but not the injection pressure constraint. These scenarios are only simulated with the horizontal well model because the injection pressure is too high for the vertical well. Alternatively, the storage volume can be distributed over multiple vertical wells. The number of wells, their spatial configuration and the distance between the wells become critical design parameters for such cases, and is beyond the scope of this investigation.

**Figure 15:**
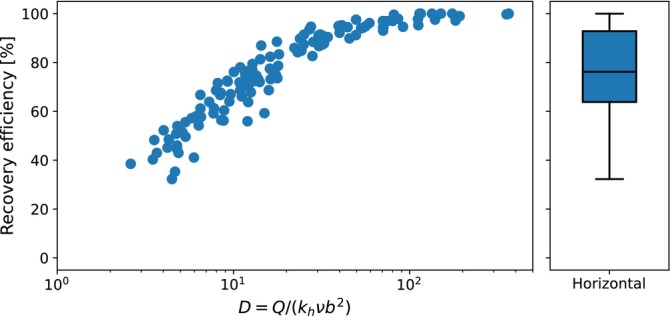
Recovery efficiency of the horizontal well simulations after five ASR cycles for the second group of parameter sets which meet the D‐value and transmissivity constraint, but not the injection pressure constraint. Recovery efficiency is shown as a function of parameter D and as a boxplot.

The recovery efficiency after five ASR cycles is shown in Figure 15 as a function of parameter D as well as a boxplot. The median D‐value of this group of parameter sets is 14.3 (25th percentile = 8.5; 75th percentile = 39.9), which is higher than the median D‐value of 4.6 in the first group (25th percentile = 2.6; 75th percentile = 10.6). The median recovery efficiency is 76% with a 25th percentile of 64% and 75th percentile of 93%. This demonstrates the potential of horizontal wells to achieve high recovery efficiencies in high pressure conditions where a single vertical well is not possible.

### More Accurate Solutions

**Figure B1:**
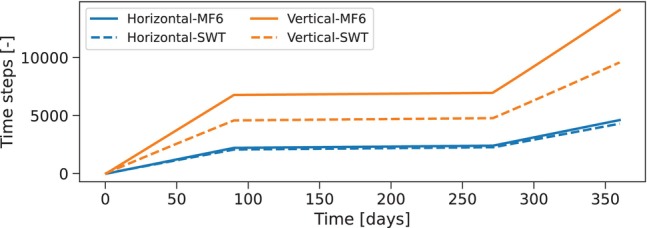
Comparison of cumulative simulation time steps over a simulation period of 360 days for the example simulation with MODFLOW 6 (Courant number = 1.4) and SEAWAT (Courant number = 0.7).

**Figure B2:**
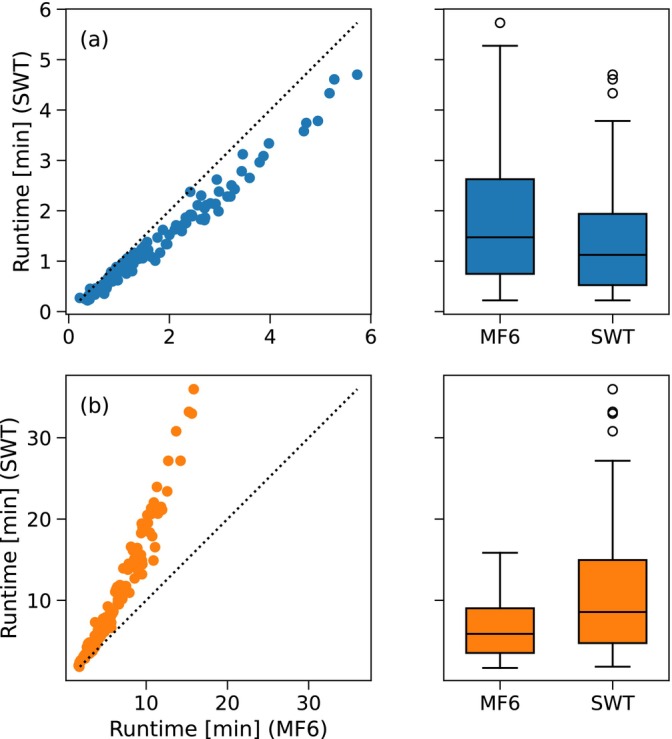
Comparison of simulation runtimes for MODFLOW 6 and SEAWAT for (a) the horizontal well and (b) the fully penetrating vertical well.

**Figure C1:**
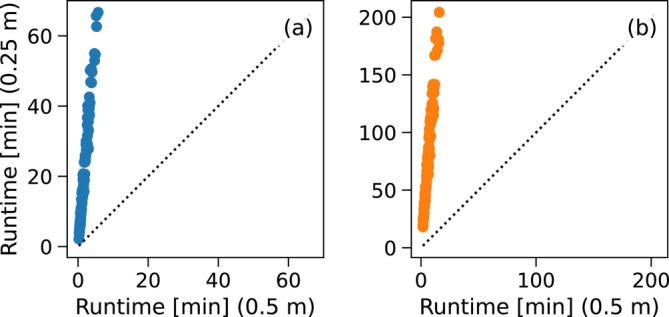
Comparison of simulation runtimes for the parameter sets of Group 1 using (a) the original grid (Δx=0.5m) and (b) the refined grid (Δx=0.25m).

The simulations of the first group of parameter sets are repeated for two additional investigations to determine whether a more accurate solution changes the recovery efficiency significantly: (i) application of a higher‐order TVD scheme for advective transport as implemented in SEAWAT, and (ii) refinement of the model grid to reduce numerical dispersion. The influence of these enhancements on the simulation results is discussed below. A detailed comparison of simulation runtimes is provided in Appendix B (Figures B1 and B2) for MODFLOW 6 and SEAWAT, and in Appendix C (Figure C1) for the varying model grid sizes.

**Figure 16:**
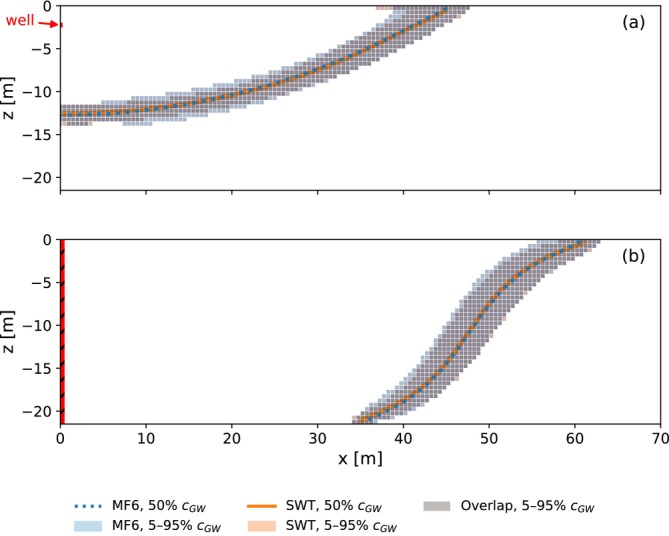
Comparison of mixing zones at the end of injection (*t* = 90 days) for second‐order TVD (MODFLOW 6) and third‐order TVD (SEAWAT) for the example simulation. (a) is the horizontal well and (b) is the fully penetrating vertical well. Shaded area shows 5%–95% concentration of background groundwater, while colored curves represent the 50% contour.

SEAWAT, Version 4 (Guo and Langevin 2002; Langevin et al. 2008) employs a third‐order TVD scheme, in contrast to the second‐order scheme employed in MODFLOW 6. SEAWAT requires some different solution parameters, which are provided in Table B1. A comparison of the mixing zones at the end of injection for the example simulation of Table 3 is given in Figure 16, for (a) the horizontal well and (b) the fully penetrating vertical well. Despite identical model parameters, the mixing zone is slightly wider in the MODFLOW 6 simulation. This is likely caused by the lower order TVD scheme in MODFLOW 6, which causes greater numerical dispersion.

**Figure 17:**
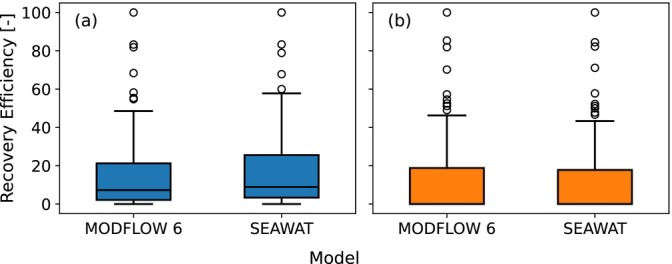
Boxplots of the recovery efficiency after one ASR cycle using second‐order TVD (MODFLOW 6) and third‐order TVD (SEAWAT). (a) is the horizontal well and (b) is the fully penetrating vertical well.

Boxplots comparing the recovery efficiencies of the MODFLOW 6 and SEAWAT simulations after one ASR cycle for the simulated parameter sets of the first group are shown in Figure 17 for (a) the horizontal well and (b) the fully penetrating vertical well. For the horizontal well, the median and 75th percentile increase slightly from 7% and 22% with MODFLOW 6 to 9% and 26% with SEAWAT. For the vertical well, the median stays constant at 0% and the 75th percentile decreases slightly from 19% to 18%. The differences in recovery efficiency are small and don't influence the conclusions of this investigation.

**Figure 18:**
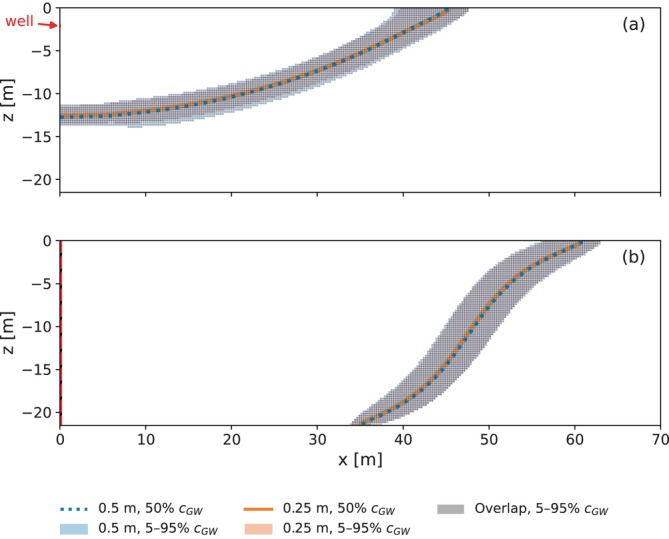
Comparison of mixing zones at the end of injection (t = 90 days) for the original grid (Δx=0.5m) and the refined grid (Δx=0.25m) for the example simulation. (a) is the horizontal well and (b) is the fully penetrating vertical well. Shaded area shows 5%–95% concentration of background groundwater, while colored curves represent the 50% contour. For the comparison the concentration data from the original grid is re‐projected onto the refined grid.

Next, the first ASR cycle is repeated with smaller cell sizes; both simulations are conducted with MODFLOW 6. The general model setup for the refined model grid investigation remains as described previously, with cell dimensions reduced from Δx=Δr=Δz=0.5m to 0.25m in the well domain. No other model parameters changed. Using a refined grid reduces numerical dispersion and improves accuracy, particularly in simulating relatively sharp concentration fronts. A comparison of the mixing zones for the original and the refined grids for the example simulation of Table 3 is given in Figure 18 for (a) the horizontal well and (b) the fully penetrating vertical well. For consistency, concentration data from the original grid is re‐projected onto the refined grid. The mixing zone is slightly wider with the original (coarser) grid due to increased numerical dispersion.

**Figure 19:**
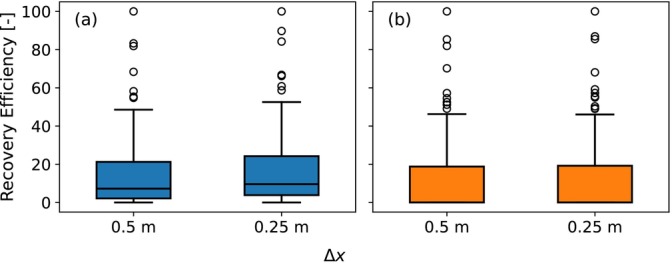
Boxplots of the recovery efficiency after one ASR cycle with the original grid (Δx=0.5m) and the refined grid (Δx=0.25m). (a) is the horizontal well and (b) is the fully penetrating vertical well.

Boxplots comparing recovery efficiencies of the different model grids after one ASR cycle for the simulated parameter sets of the first group are shown in Figure 19 for (a) the horizontal well and (b) the fully penetrating vertical well. For the horizontal well, the median and 75th percentile increase from 7% and 21% with Δx=0.5m to 10% and 24% with Δx=0.25m. For the vertical well, the median and 75th percentile stay at 0% and 19%, respectively. These differences are minor and do not affect the overall conclusions of this study.

## Conclusions

This study investigated the potential of horizontal wells for ASR in saline, low‐transmissivity environments where vertical wells are typically ineffective. The objective was to assess whether horizontal wells can achieve operationally feasible recovery efficiencies under these conditions and to determine the circumstances under which they may offer advantages over conventional vertical wells.

Numerical simulations showed that horizontal wells generally achieved significantly higher recovery efficiencies than vertical wells across the range of investigated conditions. The performance differences were primarily governed by density‐driven flow and dispersive mixing, which influence the movement and stability of the injected freshwater bubble. After five ASR cycles, horizontal wells yielded a median recovery efficiency of 45%, compared to 6% and 16% for fully and partially penetrating vertical wells, respectively. These are conservative values; higher values are obtained for shorter storage durations and/or a higher $C_{\mathrm{MAX}}$, allowing for longer extraction. For agricultural use, even fairly low efficiencies can have a significant effect on crop production and improve water security in regions facing seasonal shortages. The advantage of horizontal wells was most pronounced under strong buoyancy conditions (D < 4), where horizontal wells recovered a median of 33% while vertical wells mostly failed to recover any freshwater. However, increased dispersive mixing reduces horizontal well performance by accelerating saltwater breakthrough while modestly improving vertical well recovery by stabilizing flow near the fresh–saltwater interface. Groundwater salinity plays a dual role, influencing both buoyancy and mixing. This introduces greater variability in horizontal well performance compared to vertical wells. It was also demonstrated that horizontal wells can achieve high recovery efficiencies under high‐pressure conditions, offering a viable alternative when vertical wells are constrained by injection pressure limits.

These results underscore the potential of horizontal wells to extend ASR to hydrogeologically challenging settings, particularly saline aquifers with low transmissivity. It is recommended to consider horizontal wells in the planning of ASR systems when the hydraulic conditions require multiple vertical wells, and when buoyancy‐driven flow significantly limits vertical well performance—especially under strong buoyancy (D < 4) and generally for D < 10. Despite these advantages, horizontal wells remain more technically complex and costly to construct, which presently limits their widespread implementation. Moreover, their sensitivity to groundwater salinity demands precise design and adaptation of the well orientation, depth, and length to account for spatial salinity variability.

This study used idealized, two‐dimensional models to develop a generalized understanding of horizontal well performance in ASR systems. As such, it does not account for several site‐specific factors that can influence recovery efficiency, including non‐uniform flow along the horizontal well, background groundwater flow, aquifer heterogeneity, and vertical leakage through confining layers. The modeling framework was limited to a simplified representation of multiple partially penetrating wells. Future investigations may incorporate fully three‐dimensional representations of horizontal wells to capture the full flow dynamics and explore the effects of these complexities. Further research into optimized configurations of multiple vertical wells, including well spacing and placement strategies, will also provide valuable comparisons for ASR system design.

## Author's Note

The author does not have any conflicts of interest or financial disclosures to report.

## Data Availability

The code for groundwater model development, running, and post‐processing, as well as generation of parameter sets and schematics in this publication can be accessed at https://doi.org/10.5281/zenodo.17724838.

## Appendix B Comparison of Second and Third‐Order TVD

Results of MODFLOW 6 (MF6) to simulate relatively sharp concentration gradients are compared to SEAWAT for the first group of parameter sets for one ASR cycle. SEAWAT (SWT), Version 4 (Guo and Langevin 2002, Langevin et al. 2008) integrates a modified version of MODFLOW‐2000 (Harbaugh et al. 2000) which incorporates density‐dependent groundwater flow with the MT3DMS solute‐transport code (Zheng and Wang 1999). Table B1 summarizes the solution parameters used in the SEAWAT simulations. The corresponding parameters for the MODFLOW 6 simulations are given in Table 2. All other model parameters are the same. The vertical well is simulated with the MODFLOW Multi‐Node‐Well1‐package (MNW1) (Halford and Hanson 2002).

**Table B1:** Model Parameters Used in SEAWAT

Flow Solver Settings	Symbol	Value
Flow solver	—	Preconditioned conjugate‐gradient method (PCG)
Maximum outer iterations	mxiter (PCG)	30
Maximum inner iterations	iter1 (PCG)	100
Head change convergence criterion	hclose (PCG)	$1\times 10^{-4}$ m
Residual convergence criterion	rclose (PCG)	$1\times 10^{-4}$ m
Relaxation factor	relax (PCG)	0.97
**Transport Solver Settings**		
Transport solver	–	Generalized conjugate gradient method (GCG)
Maximum outer iterations	mxiter (GCG)	1
Maximum inner iterations	iter1 (GCG)	100
Concentration convergence criterion	cclose (GCG)	$1\times 10^{-4}$ m
**Advection Solver Settings**		
Advection solver	mixelm	−1 (TVD)
Courant number	percel	0.7

### Adaptive Time Stepping

Adaptive time stepping is employed both in MODFLOW 6 and in SEAWAT to dynamically adjust the time step size. In SEAWAT, a Courant number of 0.7 is used. The algorithm to compute the time step based on the Courant number is different in MODFLOW 6 (Version 6.6.1). Through trial and error, a Courant number of 1.4 in MODFLOW 6 was found to yield a comparable number of transport time steps for the horizontal well model. Note that SEAWAT is based on an explicit formulation, so that a low Courant number is required for stability, while MODFLOW 6 applies an implicit formulation which theoretically does not suffer from stability issues, but the time step size still affects numerical dispersion.

A comparison of the cumulative number of transport time steps required for a simulation period of 360 days for MODFLOW 6 and SEAWAT for the example of Table 3 is shown in Figure B1. For both the horizontal and vertical well model, the number of time steps increases more rapidly during the injection (0–90 days) and extraction (270–360 days) periods due to the enhanced flow induced by pumping. The lower maximum cell‐by‐cell flow rate results in larger time steps and, consequently, fewer total time steps compared to the vertical well model. For the horizontal well model, the cumulative number of transport time steps is 4,613 with MODFLOW 6 and 4,300 with SEAWAT. The discrepancy is more pronounced in the vertical well model, with MODFLOW 6 using 14,103 time steps compared to 9,582 in SEAWAT.

It is important to note that in SEAWAT, adaptive time stepping is applied exclusively to the transport process, while the flow model operates with a fixed time step defined by the user (in this case Δt=1day). The flow solution is approximated to remain constant over each flow time step, and only the transport equations are solved using the variable time steps. In contrast, MODFLOW 6 enforces equal time step sizes for both flow and transport, meaning that both components are solved for the adaptively determined time steps.

### Simulation Runtime

A comparison of runtimes for MODFLOW 6 and SEAWAT for (a) the horizontal well and (b) the fully penetrating vertical well simulations is shown in Figure B2 for the parameter sets of the first group. For the horizontal well, SEAWAT is consistently faster than MODFLOW 6. The median and 75th percentile runtimes are 1 min 9 s and 1 min 58 s for SEAWAT, compared to 1 min 29 s and 2 min 38 s for MODFLOW 6. The difference in runtime increases for simulations with longer runtimes. The shorter runtimes in SEAWAT may be attributed to its more streamlined implementation of adaptive time stepping and differences in the underlying numerical solvers.

In contrast, MODFLOW 6 is faster than SEAWAT in all vertical well simulations, despite requiring a greater number of time steps (see Figure B1). The median and 75th percentile runtimes are 5 min 55 s and 9 min 8 s for MODFLOW 6, compared to 8 min 56 s and 15 min 3 s for SEAWAT. Again, the runtime difference increases with simulation duration. A likely explanation is that the MAW‐package in MODFLOW 6 is more efficient than the MNW1‐package used in SEAWAT.

The MAW‐ and MNW1‐packages used in the vertical well model introduce greater numerical complexity compared to the WEL‐package employed in the horizontal well model. This likely accounts for the fourfold increase in MODFLOW 6 runtimes and the sevenfold increase in SEAWAT runtimes, when comparing the horizontal and vertical well models, despite the same number of model cells. Additionally, the higher discharge rate in the vertical well model increases the number of time steps (see Figure B1), further contributing to the overall computational demand.

## Appendix C Model Grid Size

The impact of grid resolution on model results is investigated. Simulations for the first group of parameter sets are repeated for one ASR cycle using a finer spatial resolution to potentially reduce numerical dispersion. The cell dimensions near the well are reduced from Δx=Δr=Δz=0.5m to 0.25m. All other model parameters are kept as before.

A comparison of runtimes for the two grid resolutions for (a) the horizontal well and (b) the vertical well simulations is shown in Figure C1. For the horizontal well, the median runtime increases from 1 min 28 s to 16 min 45 s and for the vertical well from 5 min 51 s to 1 h 12 min 54 s. This increase is primarily due to the higher number of model cells—rising from 12,642 to 43,688. Additionally, the finer grid resolution leads to smaller time steps under adaptive time stepping, thereby increasing the total number of time steps required.
